# Social Experience Interacts with Serotonin to Affect Functional Connectivity in the Social Behavior Network following Playback of Social Vocalizations in Mice

**DOI:** 10.1523/ENEURO.0247-20.2021

**Published:** 2021-03-24

**Authors:** Christopher L. Petersen, Sarah E. D. Davis, Bhumi Patel, Laura M. Hurley

**Affiliations:** 1 Department of Biology, Indiana University Bloomington, Bloomington, IN 47405; 2 Center for the Integrative Study of Animal Behavior, Indiana University Bloomington, Bloomington, IN 47405; 3 Department of Neuroscience, Indiana University Bloomington, Bloomington, IN 47406

**Keywords:** auditory, functional connectivity, serotonin, social behavior network, social isolation, vocal processing

## Abstract

Past social experience affects the circuitry responsible for producing and interpreting current behaviors. The social behavior network (SBN) is a candidate neural ensemble to investigate the consequences of early-life social isolation. The SBN interprets and produces social behaviors, such as vocalizations, through coordinated patterns of activity (functional connectivity) between its multiple nuclei. However, the SBN is relatively unexplored with respect to murine vocal processing. The serotonergic system is sensitive to past experience and innervates many nodes of the SBN; therefore, we tested whether serotonin signaling interacts with social experience to affect patterns of immediate early gene (IEG; cFos) induction in the male SBN following playback of social vocalizations. Male mice were separated into either social housing of three mice per cage or into isolated housing at 18–24 d postnatal. After 28–30 d in housing treatment, mice were parsed into one of three drug treatment groups: control, fenfluramine (FEN; increases available serotonin), or pCPA (depletes available serotonin) and exposed to a 60-min playback of female broadband vocalizations (BBVs). FEN generally increased the number of cFos-immunoreactive (-ir) neurons within the SBN, but effects were more pronounced in socially isolated mice. Despite a generalized increase in cFos immunoreactivity, isolated mice had reduced functional connectivity, clustering, and modularity compared with socially reared mice. These results are analogous to observations of functional dysconnectivity in persons with psychopathologies and suggests that early-life social isolation modulates serotonergic regulation of social networks.

## Significance Statement

Social isolation and serotonergic signaling each modulate neural functions independently of each other. It is unknown whether these factors interact to affect activity at the level of individual nuclei, and/or functional networks. Using a vocal playback paradigm, we find that acutely increasing or systemically depleting available serotonin increased cFos-immunoreactive (-ir) neurons in the social behavior network (SBN) of mice raised in social isolation compared with their socially reared counterparts. We show for the first time that mice raised in social isolation have reduced functional connectivity in the SBN relative to socially reared mice. Importantly, network perturbations were not resolved by drug treatment in isolated mice suggesting that social experience is necessary to facilitate functional relationships in the SBN.

## Introduction

The importance of social rearing has been evident since the 1960s and the now-controversial “Harlow monkey experiments,” which demonstrated that early-life social isolation deprives macaques of the experiences necessary to develop into functional adults (Harlow et al., 1965). The ability to amicably behave and communicate with conspecifics is important to social cohesion, which in turn affects individual fitness and psychological wellness (Heim and Nemeroff, 2001; Bailey and Moore, 2018). Neural systems have evolved to support social cohesion (Goodson, 2013; Matthews and Tye, 2019) such that social interactions may carry positive valence (Goodson and Wang, 2006), and be reinforced by the brain’s reward circuitry (Dölen et al., 2013). Early-life social immersion or deprivation may shape the circuits responsible for the appropriate expression of social behaviors.

One such circuit is the vertebrate social behavior network (SBN). This includes the lateral septum (LS); bed nucleus of the stria terminalis (BNST); medial preoptic area (mPOA); paraventricular (PVN), anterior (AH), and ventromedial (VMH) hypothalamic nuclei; the ventral tegmental area (VTA); and periaqueductal gray (PAG; Newman, 1999; Goodson, 2005; Goodson and Kingsbury, 2013). Rather than an exhaustive list of regions that facilitate social processes (Rogers-Carter and Christianson, 2019), the SBN is an evolutionarily conserved suite of nuclei responsible for interpreting and generating responses to social stimuli (Goodson, 2005; O’Connell and Hofmann, 2012). SBN nuclei or “nodes” are differentially engaged during specific behaviors (Lin et al., 2011; Lee et al., 2014; Decot et al., 2017; McHenry et al., 2017; Kohl et al., 2018; Tschida et al., 2019); however, coordinated patterns of activity or functional connectivity (Friston, 2011) across the SBN contribute to variation in behavioral output (Goodson, 2005; Goodson and Kabelik, 2009).

Since the original proposal of the SBN (Newman, 1999), analytical tools have been developed to quantitatively describe functional anatomic networks (Sporns, 2010; Fornito et al., 2016). For example, metrics such as density measure functional connectivity in a given network, whereas the clustering coefficient is an indicator of “small-world” networks which display increased efficacy of communication among regions (Watts and Strogatz, 1998). Community structure/modularity calculates the degree to which nodes assemble into functionally similar clusters (Newman and Girvan, 2004). Functional networks are disrupted in human psychopathologies (Bullmore et al., 1997; van den Heuvel and Sporns, 2019), emphasizing the importance of investigating network-level features in rodent translational models (Van den Heuvel et al., 2016). Network-based analyses in non-traditional model systems describe changes in SBN functional connectivity following presentation of socially salient vocal stimuli (Hoke et al., 2005; Ghahramani et al., 2018); however, no such studies exist in laboratory mice.

Murine vocalizations are a source of context-dependent information during social interactions (Hanson and Hurley, 2012; Finton et al., 2017; Warren et al., 2018, 2020; Sangiamo et al., 2020). Vocal processing relies on auditory circuitry as well as functionally diverse nuclei such as the SBN. For example, receivers must extract the physical characteristics of vocal signals (e.g., frequency, duration, amplitude, etc.) and interpret them in light of their own experiences and current conditions (Petersen and Hurley, 2017). Investigating whether social isolation disrupts vocal processing in circuits such as the SBN will be important in understanding the mechanisms underlying aberrant behavior in mouse models of communicative and affective disorders (Portfors and Perkel, 2014).

Serotonergic signaling is sensitive to social isolation: socially isolated mice downregulate 5-HT receptor expression in hypothalamic nodes of the SBN (Schiller et al., 2003; Bibancos et al., 2007). As anatomically distinct regions of the dorsal raphe nucleus send serotonergic projections to the SBN (Schwarz et al., 2015; Muzerelle et al., 2016; Beier et al., 2019), serotonin may modulate SBN activity in accordance with an animal’s internal state and changes in the external environment (Muzerelle et al., 2016; Niederkofler et al., 2016; Ren et al., 2018). Broadly activating serotonergic pathways affects neural activity markers across a distributed suite of nuclei including the SBN (Giorgi et al., 2017; Grandjean et al., 2019); however, it remains unknown whether social experience interacts with serotonin signaling to affect activity-dependent measures and network-level metrics such as functional connectivity.

We use immediate early gene (IEG) mapping to test the hypothesis that serotonin signaling interacts with social experience to affect patterns of cFos-immunoreactive (-ir) neurons in the SBN of male mice following presentation of female broadband vocalizations (BBVs). Increasing available serotonin increased the IEG response in several SBN nodes. This effect was more prominent in socially isolated mice regardless of drug treatment. Despite increases in cFos-ir neurons, network analyses reveal fewer functional relationships within the SBN of socially isolated mice.

## Materials and Methods

### Animal information

**Figure 1.**
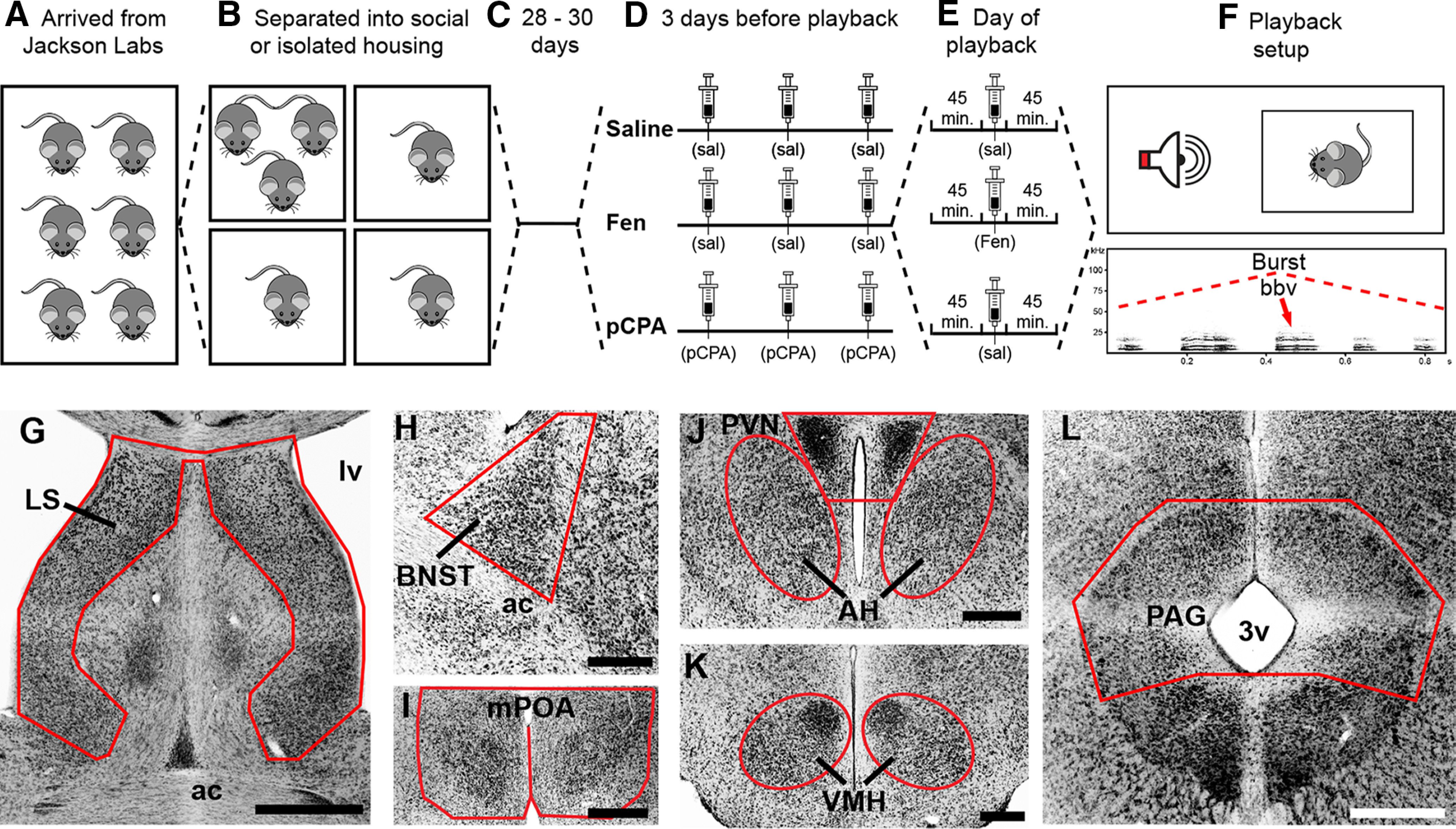
Experimental design: playback paradigm and neuroanatomy. ***A***, Male CBA/J mice arrived from The Jackson Laboratory at 18–24 d postnatal and were immediately separated into social (three per cage) or isolated (one per cage) housing (***B***). ***C***, Mice remained in their respective housing conditions for 28–30 d. ***D***, SAL and FEN mice received SAL injections for 3 d before playback; mice in the pCPA group received pCPA injections on these days. ***E***, Forty-five minutes before playback trials, mice in the SAL and pCPA group received SAL injections, whereas FEN mice received FEN. ***F***, Playback trials were 60 min and consisted of 14–15 bursts of five female BBVs. ***G–L***, Representative inverse fluorescent 10× photomicrographs showing seven nodes of the SBN: LS (***G***), BNST (***H***), mPOA (***I***), PVN and AH (***J***), VMH (***K***), and PAG **(*L***). ac, anterior commissure; lv, lateral ventricle; 3v, third ventricle. Scale bars: 1 mm (***G***) and 500 μm (***H–L***).

The Indiana University, Bloomington Institutional Animal Care and Use Committee (protocol #15-021) approved all of the following experiments. Individual cohorts of male CBA/J mice (*Mus musculus)* from different litters were shipped from The Jackson Laboratory and received at 18–24 d of age (Fig. 1*A*). Each cohort was assigned to 1 of three pharmacological treatment groups: saline (SAL; control), fenfluramine (FEN), or pCPA (see pharmacological details below). Upon arrival, mice were separated into either social housing of three mice per cage or into isolated housing (Fig. 1*B*). Mice remained in social (SOC) or isolated (ISO) conditions on a 14/10 h light/dark cycle with *ad libitum* access to food and water and weekly cage changes for 28–30 d before vocal playback (Fig. 1*C*,*D*). ISO mice were physically separated from conspecifics; however, all experimental animals were housed in the same room within our vivarium. While ISO mice were potentially exposed to olfactory, auditory, and/or visual stimuli from neighboring cages, similar conditions did not attenuate the effects of social isolation in other studies (Keesom et al., 2017a,b; Manouze et al., 2019).

### Pharmacology

pCPA methyl ester hydrochloride (pCPA; 4-chloro-DL-phenylalanine methyl ester hydrochloride; Tocris) was used to deplete systemic serotonin. pCPA is a non-reversible inhibitor of tryptophan hydroxylase (TPH; the rate-limiting enzyme in serotonin synthesis; Koe et al., 1966). Over the course of 3–4 d, pCPA depletes serotonin in brain regions including the hippocampus, striatum, and cortex (Dailly et al., 2006). Conversely, dexfenfluramine hydrochloride (Fenluramine; (S)-N-ethyl-α-methyl-3-(trifluoromethyl) benzeneethanamine hydrochloride; Tocris), which releases stores of vesicular serotonin and blocks its reuptake at the synapse (Davis and Faulds, 1996; Rothman and Baumann, 2002), was used to acutely increase levels of available serotonin.

FEN and pCPA were diluted in 0.9% sterile SAL within 3 d of use. pCPA was administered at 200 mg/kg in a volume of 5 ml/kg; FEN was administered at 100 mg/kg in a volume of 5 ml/kg (Hanson and Hurley, 2016). Sterile SAL (vehicle; 10 ml/kg) was used for all control injections. Each mouse in SOC cages received the same pharmacological treatment. Injections were administered interperitoneally following brief anesthetization with isoflurane, after which mice were returned to their home cage. Beginning 3 d before playback, mice were transferred from housing quarters to the experimental room where they received injections at roughly 24-h intervals in the morning. Over the course of these 3 d, pCPA mice received pCPA injections while FEN and SAL mice received equivalent injections of sterile SAL (Fig. 1*D*). Following injections, mice remained in the experimental room for 45 min before being returned to the vivarium. This process was designed to habituate mice to injections and being moved between rooms to reduce non-specific cFos expression before playback trials. On the day of playback, pCPA and SAL mice were injected with sterile SAL 45 min before trials. FEN mice received FEN injections 45 min before playback (Fig. 1*E*). For each treatment group *n* = 9 except for SOC-SAL, where *n* = 8. Over the course of injections, ISO-pCPA mice lost a significant amount of weight (paired *t*_(8)_ = 2.82, *p* < 0.05, mean difference 0.43 ± 0.15 g), but there was no difference between the weights of treatment groups on the day of playback (*p* = 0.2).

### Playback trials

Ninety minutes before playback trials, mice were retrieved from animal quarters and placed in a quiet room. After 45 min, the first of three animals received an injection (as above) and was returned to its home cage (Fig. 1*E*). Forty-five minutes after injection, focal mice were transferred from their home cage to an identical testing cage (12 × 6 × 6 inches) with fresh bedding within in a sound attenuation chamber (Coulbourn Habitest) with an ultrasonic speaker (Ultrasonic Dynamic Speaker Vifa, Avisoft Bioacoustics) powered by an UltraSoundGate Player 116 (Avisoft Bioacoustics). Trials were monitored with a CCD video camera (30 fps) placed above the test cage, with SuperDVR software (Q-See, Digital Peripheral Solutions Inc.) and a Q-see four channel DVR PCI video capture card. Trials were 60 min and playback consisted of 14–15 naturalistic bursts of five female BBVs (Fig. 1*F*), the final number of BBVs (70–75) represented the average number of BBVs emitted during the study from which they were recorded. All mice were played back the same BBV sound file; omission of the final burst of five BBVs was counterbalanced across groups. Following trials, spectrograms of the playback were created in Avisoft, and the number of male-emitted ultrasonic vocalizations (USVs) were quantified.

### Playback generation

Source BBVs were originally recorded during sociosexual interactions between male and female CBA/J mice. First, spectrograms of sociosexual interactions were generated using Avisoft SASlab Pro software; next, individual female BBVs were located, high-pass filtered to remove any potentially overlapping male USVs, and copied into a new playback audio file. We assembled naturalistic bursts of five individual BBVs and interspersed 270 s of silence between bursts. BBVs were calibrated by matching rms intensity of the playback (as recorded in the testing arena) to the intensity of the originally recorded vocalizations. The same condenser microphone (CM16/CMPA, Avisoft Bioacoustics) with an UltraSoundGate 116Hb sound card (250-kHz sample rate Avisoft Bioacoustics) was used to assess the intensities of the originally recorded vocalizations and the playback.

In naturalistic social interactions, female BBVs correlate with male-directed aggression (i.e., rejection-like behaviors), as they also emitted during mounting, BBVs are considered to be functionally ambiguous (Finton et al., 2017). Our playback file consisted of BBVs that were acquired during multiple interactions where mounting either did or did not occur. Thus, any potential structural differences in BBVs emitted during different contexts (i.e., mounting vs rejection) would not shape the overall valence of playback.

### Immunohistochemistry (IHC)

Playback lasted for 60 min, at which point focal animals remained in the sound attenuation chamber for 30 additional minutes to allow for accumulation of the cFos protein (Kovacs, 2008). Ninety minutes following the onset of playback, mice were deeply anesthetized with isoflurane and transcardially perfused with ice-cold Krebs-Henseleit solution (pH 7.2) followed by 50 ml of 4% paraformaldehyde in phosphate buffer (PFA). Brains were extracted and postfixed overnight in PFA, transferred to 30% sucrose in PBS (pH 7.4) for ∼48 h, and cut into three series of 50-μm sections in the coronal plane using a freezing microtome. Sections were collected throughout the rostral-to-caudal extent of the inferior colliculus (IC; approximately bregma −5.34 thru −4.36 mm) and starting at the appearance of the median eminence (bregma −1.94 mm) through the bifurcation of the anterior commissure (AC; ∼ bregma +0.38 mm). Sections were stored in cryoprotectant solution at −80°C until IHC. Three separate IHC runs counterbalanced across treatment groups were performed as follows.

Tissue was equilibrated to room temperature; free-floating sections were first sorted in PBS, then washed for 5 × 5 min in PBS, followed by a 1-h soak in blocking solution: PBS + 10% donkey serum (DS; Millipore) and 0.3% Triton X-100 (Tx; Millipore). Sections were incubated for 18 h at room temperature in rabbit anti-cFos (F7799, lot:105M4831V; Sigma-Aldrich) diluted 1:2000 in PBS + 5% DS and 0.3% Tx (diluent). Following primary incubation, sections were washed 4 × 5 min in PBS + 0.5% DS, and then incubated for 2 h at room temperature in Alexa Fluor donkey anti-rabbit 680 (Life Technologies) diluted 6:1000 in diluent. Sections were washed 3 × 10 min in PBS, followed by a 30 min incubation in NeuroTrace 500/525 Green Fluorescent Nissl (NT) diluted 1:200 in PBS. Following a final, 10-min PBS wash, sections were mounted onto chrome alum-subbed slides and cover-slipped with Prolong Gold Antifade reagent with 4,6-diamidino-2-phenylindole (DAPI; Life Technologies) and stored at 4°C until microscopy.

### Image acquisition and anatomy

All images were collected at 10× with 568-nm resolution using a Leica SP8 scanning confocal microscope. cFos-ir neurons were visualized using a 680-nm laser line; DAPI and NT were visualized using 405- and 490-nm laser lines, respectively. The intensity of each laser line was identical for all images, and tissue was scanned at 12 separate z planes spaced 2.41 μm apart. When more than one confocal image was needed to capture the expanse of a nucleus (LS, mPOA, VMH, PAG) or adjacent nuclei (i.e., PVN and AH), images were automatically merged using Leica Application Suite X software (Leica Microsystems). In instances where tissue was damaged, the next available section was used.

SBN regions were identified based on cytoarchitecture at approximate rostral-caudal levels relative to bregma as per the mouse brain atlas (Paxinos and Franklin, 2004). The LS (Fig. 1*G*) was collected beginning at the bifurcation of the AC (bregma 0.38 mm) and continued for three consecutive sections. We captured the medial division of the BNST starting at the level where the bifurcation of AC begins to close (bregma 0.26 mm). BNST (Fig. 1*H*) was sampled bilaterally in two consecutive sections within boundaries established by the lateral ventricle and the stria terminalis (lateral), the fornix (medial), and ventrally by the AC. Sampling for mPOA (Fig. 1*I*) began at the closure of AC (bregma 0.14 mm) and continued for two consecutive sections. PVN was shot at three consecutive sections beginning at the appearance of a triangular cluster of neurons immediately adjacent to the dorsal aspect of the third ventricle (bregma −0.70 mm). We collected AH beginning at the second level of PVN (bregma −0.82 mm; Fig. 1*J*) and continued for three consecutive sections. The final level of AH overlapped with the first section where sampling for VMH began (bregma −1.22) and continued for two to three sections (Fig. 1*K*). PAG (Fig. 1*L*) was collected for three consecutive sections beginning approximately at bregma −4.24 mm.

Z stacks for laser lines 405 (DAPI), 490 (NT), and 680 (cFos-ir) were projected using the maximal intensity function in Fiji (National Institutes of Health; Schindelin et al., 2012), and saved as .tif files before analysis. A single observer blind to animal identity and treatment group performed all image analyses and microscopy.

### Cell counting

Regions of interest (ROIs) were drawn around the boundaries of SBN nodes based on cytoarchitecture (Fig. 1*G–L*) and saved using the Fiji ROI manager. cFos-ir neurons were quantified using custom macros in ImageJ as follows. First, background was subtracted from cFos images using the rolling ball function with a radius of 50 pixels. ROIs derived from NT images were transferred to the corresponding cFos channel. Using the internal clipboard function, we created a new image containing only the selected ROI. Tsai’s moments threshold was applied using Fiji’s Auto Threshold plugin v1.17. The thresholded image was then made binary, the watershed function was applied, and the analyze particles function was run thresholding out objects with fewer than 75 pixels and a circularity <0.15. Cell counts were normalized by multiplying the total number of cFos-ir neurons in each region by 100 divided by the sum of the areas of the ROI(s) from which they were obtained.

### Statistics

Inferential statistics were performed in JMP Pro version 14 (SAS Institute Cary) with an α = 0.05, or GraphPad Prism version 8. We used repeated measures multivariate ANOVA (MANOVA) to test the between-subject effects of housing (SOC vs ISO) and drug treatment (SAL vs FEN vs pCPA) on the within-subjects measure of cFos-ir neurons/100 μm^2^ across seven nodes of the SBN. We found main effects of housing and drug treatment, as well as a significant housing-by-drug interaction (see Results). We followed MANOVA with a series of linear mixed model analyses with housing and drug treatment as fixed effects to test for group differences in cFos-ir neurons within each SBN node. Our model included IHC run as a random effect to control for potential variation introduced by separate IHC procedures. As SOC mice were housed in groups of 3, we also included cage as a random effect to control for potential within-cage influence on cFos expression. *Post hoc* differences between groups were assessed via independent *t* tests where applicable.

Next, we performed pair-wise correlations on the number of cFos-ir neurons to test for functional relationships between SBN nodes within each of our six treatment groups; *p* values obtained from Pearson coefficients were corrected for multiple comparisons using the two-stage linear step-up procedure of Benjamini, Krieger, and Yekutieli (Benjamini et al., 2006). To test for differences in the distribution of internodal correlations between groups, we performed principal components analysis (PCA) on the covariation matrix derived from these data.

Network analyses were performed on within-group correlations using Gephi open source network analysis and visualization software version 0.9.2 (Bastian et al., 2009). Functional relationships were visualized as unweighted, undirected graphs using the ForceAtlas2 algorithm, which spatially distributes nodes based on the overall strengths of each node’s correlations (Bastian et al., 2009; Jacomy et al., 2014). Graphs were subsequently filtered so that non-significant edges (i.e., correlations *p* > 0.05) were excluded from visualization. The overall relatedness of any given region is indicated not only by its shared edges, but by its position relative to other nodes. In order to quantitatively describe functional relationships among groups, we performed three separate graph analyses in Gephi. First, we calculated network density, the number of significant intraregional correlations as a proportion of the total number of possible correlations, for each treatment group. This analysis, a graph-based supplement to our strength of correlation analysis (see Results), quantifies the overall functional connectivity of the SBN in each treatment group. Next, for each treatment group we calculated the average clustering coefficient as the likelihood for any pair of a node’s functional connected neighbors to be connected to each other (Watts and Strogatz, 1998; Fornito et al., 2016). Finally, we performed community analysis which parses nodes into highly interconnected subgroups which is indicative of functional commonality (Fornito et al., 2016).

## Results

Repeated measures MANOVA found significant effects of housing (*F*_(6,36)_ = 4.45, *p* = 0.002) and drug treatment (*F*_(12,74)_ = 5.71, *p* < 0.0001), as well as a significant housing-by-drug interaction (*F*_(12,74)_ = 3.17, *p* = 0.001) on number of cFos-ir neurons/100 μm^2^ across seven nodes of the SBN. Next, we performed linear mixed model analyses with housing and drug treatment as fixed factors, controlling for random effects of IHC run and cage. Below we report only *p* values for linear mixed models; a complete summary can be found in Table 1. Arithmetic means for housing and drug treatments are reported in Table 2. Results from the applicable *post hoc* comparisons can be found in Extended Data Figure 2-1.

**Table 1:** Summary of mixed linear models

Source	DF	*F*	*p*	Variance component	Estimate	SE	95% lower	95% upper	*p*
LS									
Housing	1, 22.8	0.28	0.60	Cage number	−4.18	4.95	−13.89	5.53	0.40
Drug	2, 22.8	15.98	<0.0001***	IHC run	7.83	9.45	−10.70	26.36	0.41
Housing × drug	2, 22.8	4.84	0.02*	Residual	33.51	8.33	21.73	58.40	
BNST									
Housing	1, 14.5	7.23	0.02*	Cage number	3.15	6.84	−10.25	16.55	0.65
Drug	2, 14.5	52.63	<0.0001***	IHC run	2.80	4.31	−5.64	11.24	0.52
Housing × drug	2, 14.5	0.49	0.62	Residual	20.98	6.70	12.23	44.13	
mPOA									
Housing	1, 20.5	6.67	0.02*	Cage number	57.24	24.33	9.56	104.93	0.02*
Drug	2, 20.5	25.34	<0.0001***	IHC run	34.86	40.78	−45.08	114.80	0.39
Housing × drug	2, 20.5	1.96	0.17	Residual	28.66	10.54	15.59	69.15	
PVN									
Housing	1, 19.1	10.12	0.005**	Cage number	21.13	12.54	−3.44	45.70	0.09
Drug	2, 19.1	40.99	<0.0001***	IHC run	13.95	17.01	−19.39	47.29	0.41
Housing × drug	2, 19.1	8.25	0.003**	Residual	25.61	8.42	14.71	55.33	
AH									
Housing	1, 21.2	0.18	0.67	Cage number	0.11	4.80	−9.29	9.51	0.98
Drug	2, 21.2	6.60	0.001**	IHC run	4.92	6.33	−7.48	17.32	0.44
Housing × drug	2, 21.2	1.91	0.17	Residual	24.55	6.49	15.53	44.58	
VMH									
Housing	1, 21.5	2.52	0.13	Cage number	7.18	2.50	2.28	12.08	0.004**
Drug	2, 21.5	14.47	<0.0001***	IHC run	0.49	1.01	−1.48	2.46	0.63
Housing × drug	2, 21.5	0.06	0.94	Residual	1.48	0.56	0.79	3.68	
PAG									
Housing	1, 22.5	6.11	0.02*	Cage number	32.71	11.90	9.39	56.04	0.01**
Drug	2, 22.5	0.91	0.42	IHC run	5.48	8.02	−10.24	21.21	0.49
Housing × drug	2, 22.5	1.45	0.26	Residual	10.71	3.87	5.88	25.36	

Statistically significant main effects and interactions indicated with asterisks: *p < 0.05, **p < 0.01, ***p < 0.001.

**Table 2:** Summary of group means with housing and drug treatments

Housing	LS	BNST	mPOA	PVN	AH	VMH	PAG
Social	25.25 ± 3.8	13.6 ± 5.2	31.58 ± 6.6	15.97 ± 4.1	15.24 ± 2.0	4.48 ± 2.1	12.50 ± 0.6
Isolated	26.23 ± 2.5	17.56 ± 5.3*	40.65 ± 10.2*	23.39 ± 10.4**	16.00 ± 2.1	6.17 ± 2.1	18.42 ± 2.6*

Summary of arithmetic group means of cFos-ir neurons within drug treatments ± SE							
Drug treatment	LS	BNST	mPOA	PVN	AH	VMH	PAG
SAL	22.95 ± 1.2	10.32 ± 1.0	27.73 ± 0.1	12.71 ± 1.1	14.79 ± 1.5	3.587 ± 0.5	13.83 ± 0.9
FEN	31.36 ± 0.9**	26.0 ± 2.1***	52.59 ± 8.1***	34.2 ± 10.0***	19.06 ± 1.0*	9.458 ± 0.9***	15.2 ± 2.0
pCPA	22.9 ± 3.6	10.42 ± 2.9	28.02 ± 5.4	12.13 ± 1.0	13.02 ± 1.6	2.933 ± 1.2	17.35 ± 6.0

Values expressed as arithmetic mean ± SEM. Statistically significant differences group means indicated with asterisks: *p < 0.05, **p < 0.01, ***p < 0.001; *p* values corrected for multiple comparisons (Benjamini et al., 2006). Complete *post hoc* comparisons can be found in Extended Data Figure 2-1.

**Figure 2.**
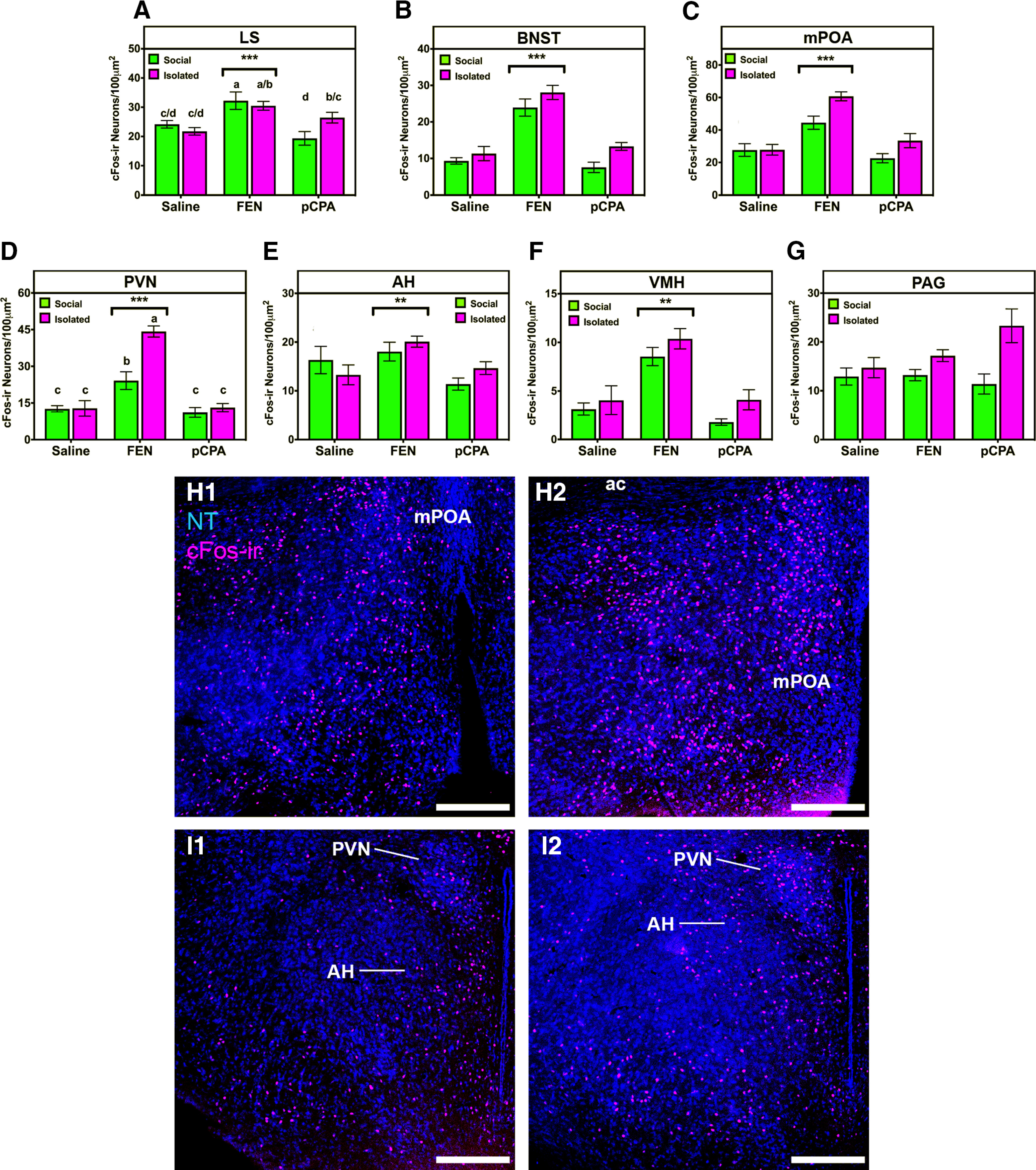
Summary linear mixed model analysis of cFos-ir within nodes of the SBN. ***A–G***, All data are represented as arithmetic mean ± SEM between socially housed mice (green) and socially isolated mice (magenta). Asterisks represent main effects of drug treatment, **p < 0.001, ***p < 0.0001. Letters indicate *post hoc* differences (independent *t* test, *p* values corrected for multiple comparisons) in the case of housing by drug interaction. ***H***, ***I***, Representative photomicrographs in mPOA (***H***) and PVN (***I***) showing the effects of FEN on cFos-ir in socially reared (***H1***/***I1***) or socially isolated (***H2***/***I2***) mice. NT, blue; cFos-ir, magenta. Scale bar: 250 μm. Please see Extended Data Figure 2-1 for a complete summary of *post hoc* analyses for linear mixed models performed within each SBN region.

10.1523/ENEURO.0247-20.2021.f2-1 Extended Data Figure 2-1 Complete summary of *post hoc* analyses for linear mixed models performed within each SBN region. Download Figure 2-1, DOCX file.

Our model detected effects of housing in the BNST (*p* = 0.017), mPOA (*p* = 0.018), PVN (*p* = 0.005), and PAG (*p* = 0.021). ISO mice had higher numbers of cFos-ir neurons in each one of these regions (Tukey’s HSD, *p* ≤ 0.02). We found significant effects of drug treatment in LS (*p* < 0.0001; Fig. 2*A*), BNST (*p* < 0.0001; Fig. 2*B*), mPOA (*p* < 0.0001; Fig. 2*C*), PVN (*p* < 0.0001; Fig. 2*D*), AH (*p* = 0.006; Fig. 2*E*), and VMH (*p* = 0.0001; Fig. 2*F*). In each region except for AH, FEN mice had significantly more cFos-ir neurons than both SAL and pCPA mice (Tukey’s HSD, *p* ≤ 0.0007). In AH, FEN mice had significantly more cFos-ir neurons than pCPA mice (Tukey’s HSD, *p* = 0.005), and a trend toward an increase relative to SAL mice (Tukey’s HSD, *p* = 0.054). Interestingly, PAG was the only region where our model did not detect a significant effect of drug treatment (*p* > 0.4; Fig. 2*G*). Drug effects were dependent on housing conditions as indicated by significant interaction terms in LS (*p* = 0.018) and PVN (*p* = 0.003). In LS, the interaction is driven by an increase in cFos-ir neurons in ISO-pCPA compared with the SOC-pCPA mice. In PVN, the interaction is driven by an almost twofold increase in cFos-ir neurons in ISO-FEN compared with SOC-FEN mice.

We found a main effect of housing (*F*_(1,47)_ = 5.97, *p* = 0.02) and a significant housing-by-drug interaction (*F*_(2,47)_ = 6.03, *p* = 0.005; data not shown) on the number of USVs emitted by focal males during playback. The interaction was driven by a significant increase in USV production in ISO-SAL compared with SOC-SAL mice (Tukey’s HSD, *p* = 0.004). There were no differences in USV production between SOC and ISO males in either the FEN or pCPA groups (*p* > 0.8). Interestingly, there were no relationship between USV production and cFos-ir within the SBN of any treatment group.

**Figure 3.**
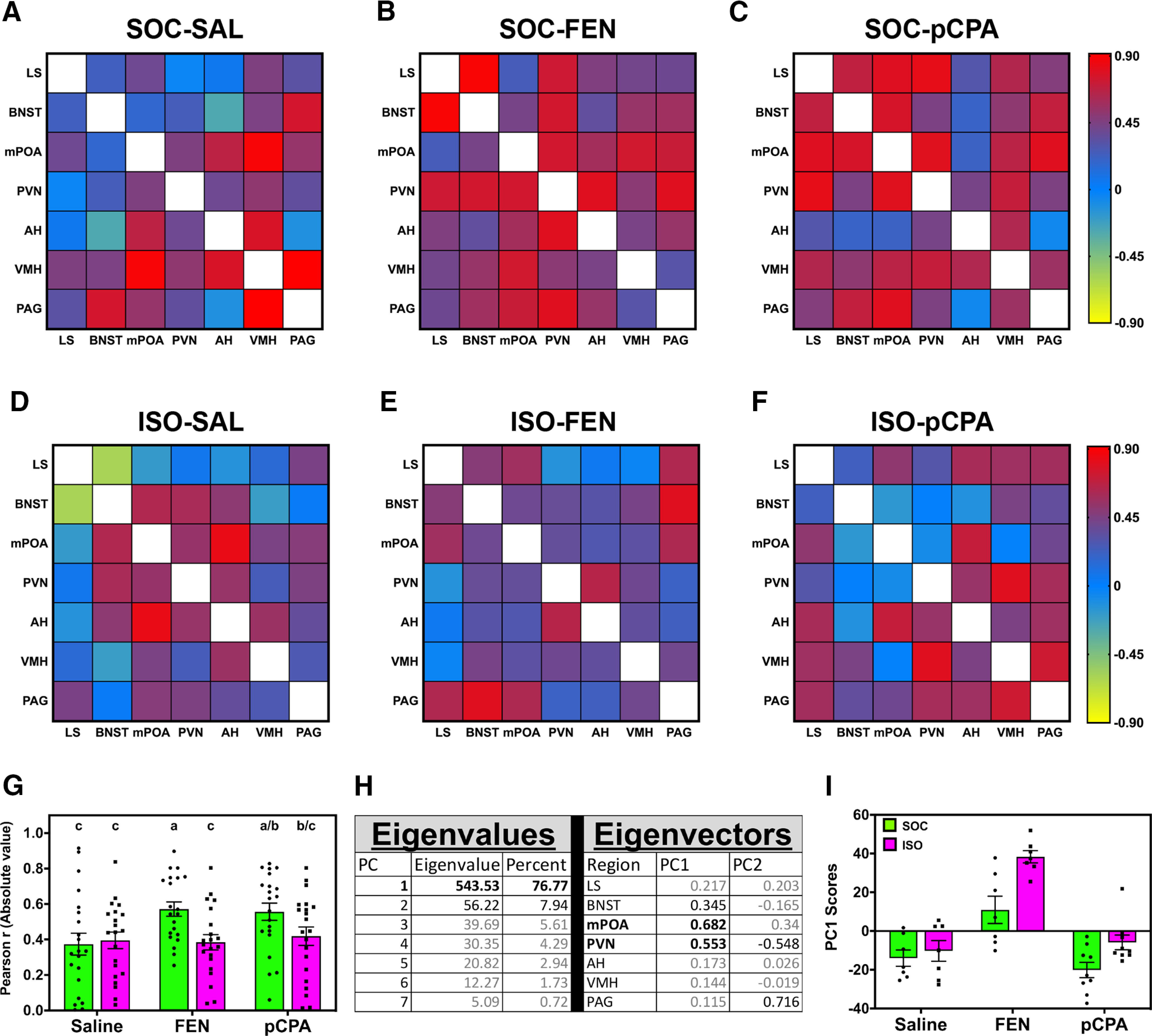
Correlated patterns of cFos-ir are dependent on social experience. ***A–F***, Heatmap matrices represent pairwise correlations between cFos-ir neurons with SBN nodes (boxes); colors indicate Pearson correlation coefficients. White boxes are self-correlations (*r* = 1); data are mirrored above and below the diagonal. ***G***, Comparison between the absolute value of Pearson correlation coefficients between groups. Data are represented as mean ± SEM; letters indicate *post hoc* differences (independent *t* test, *p* values corrected for multiple comparisons). ***H***, Results of PCA on covariation matrix derived from ***A–F***. ***I***, Distribution of PC1 scores between groups; data are represented as mean ± SEM. Please see Extended Data Figure 3-1 for a complete summary of pairwise correlation statistics.

As the SBN comprises a reciprocally connected anatomic network (Hahn et al., 2019), we tested whether correlations of neural activity markers between nodes (i.e., functional connectivity) varied between treatment groups. Figure 3 summarizes these data as heatmap matrices based on the Pearson *r* values of each pairwise correlation. A detailed summary of pairwise correlations of cFos-ir neurons between SBN nodes within each of our six treatment groups can be found in Extended Data Figure 3-1. Cursory analysis of the heatmaps suggested that SOC-FEN (Fig. 3*B*) and SOC-pCPA (Fig. 3*C*) had more relatively strong correlations (i.e., more functional connectivity) than all other treatment groups. To test this, we used two-way ANOVAs on the absolute value of Pearson *r* scores of each treatment group (Tanimizu et al., 2017). We found a main effect of housing (*F*_(1,120)_ = 6.33, *p* = 0.013; Fig. 3*G*), where SOC mice had larger Pearson *r* values than ISO mice (Tukey’s HSD, *p* = 0.013). Drug treatment (*F*_(2,120)_ = 2.68, *p* = 0.07) and housing-by-drug interaction (*F*_(2,120)_ = 2.45, *p* = 0.09) approached but did not reach statistical significance.

10.1523/ENEURO.0247-20.2021.f3-1 Extended Data Figure 3-1 Summary of pairwise correlation statistics describing the relationship between cFos-ir neurons within SBN nodes. Download Figure 3-1, DOC file.

While we did not find a statistically significant effect of drug treatment on the strength of internode correlations, we hypothesized that pharmacologically increasing or systemically depleting available serotonin would differentially affect the distribution of functional relationships. We thus performed PCA on the covariation matrix generated by cFos-ir counts. PC1 had an eigenvector of 543.53, which accounted for over 76% of the variation and contained eigenvalues most strongly loaded by mPOA (0.682) and PVN (0.553; Fig. 3*H*). We analyzed PC1 scores between groups via two-way ANOVA and found main effects of housing (*F*_(1,41)_ = 14.11, *p* < 0.001) and drug treatment (*F*_(2,41)_ = 39.28, *p* < 0.0001; Fig. 3*I*), but no significant interaction (*F*_(2,41)_ = 2.43, *p* = 0.10). FEN mice had divergent and significantly different (Tukey’s HSD, *p* < 0.0001) PC1 scores from both SAL and FEN mice; Thus, despite having similar overall Pearson *r* values, the distribution of correlations among SBN nodes was different between drug treatment groups.

**Figure 4.**
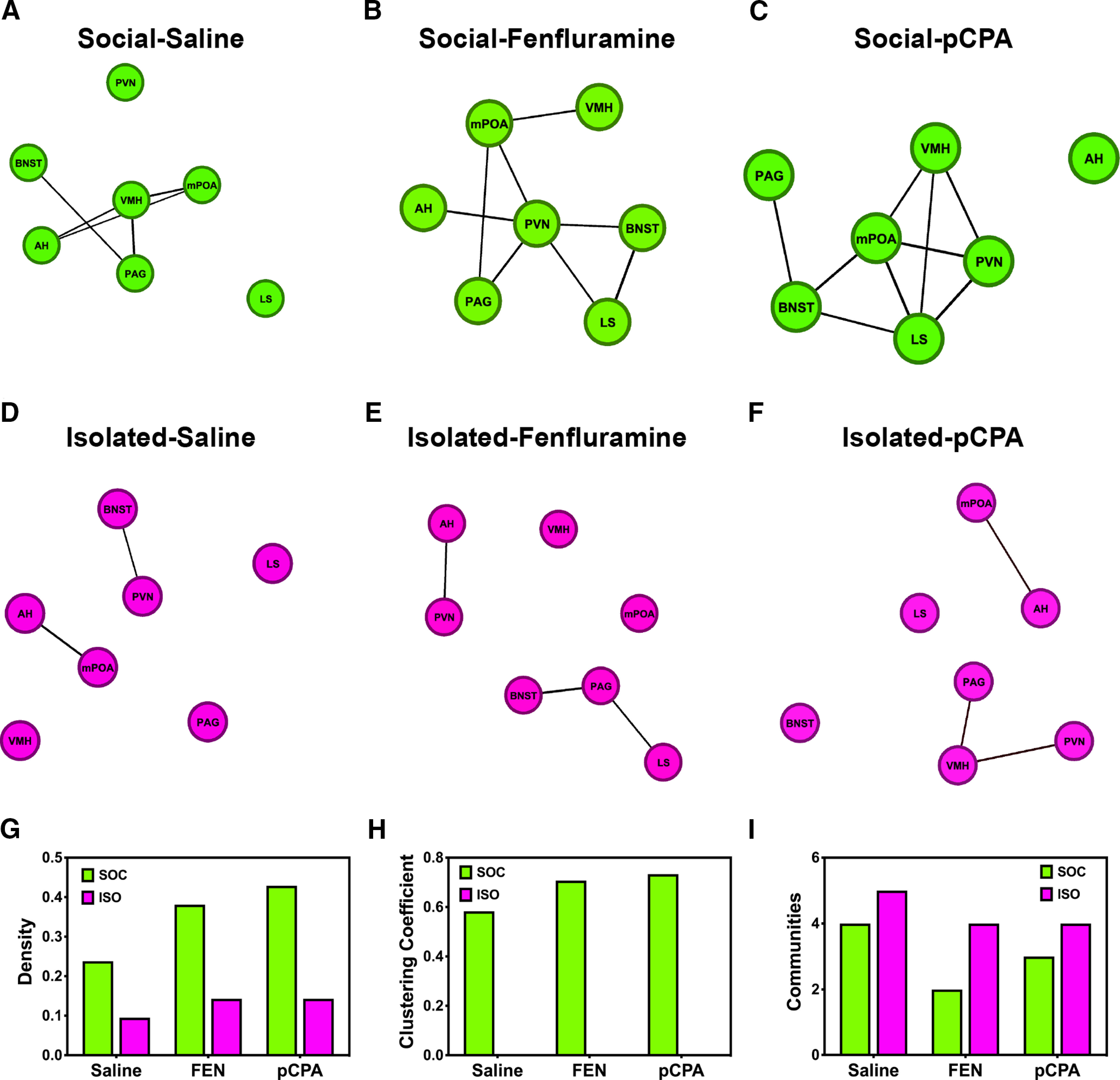
Correlated patterns of activity form different functional networks between treatment groups. ***A–F***, Individual nodes are represented as green (SOC) or magenta (ISO) circles. The spatial distribution of nodes is determined by their individual strengths of correlation (Jacomy et al., 2014). Lines (edges) connecting nodes are indicative of statistically significant Pearson *r* values (*p* < 0.05); non-significant edges (*p* > 0.05) are excluded from graphs. ***G–I***, Network measures vary between SOC and ISO mice. ***G***, SOC mice (green) have denser functional networks than ISO mice (magenta). ***H***, SOC mice have higher clustering coefficients than ISO mice, whose clustering coefficient is zero in each drug treatment group. ***I***, SOC mice formed fewer thus more densely populated functional communities than ISO mice.

We visualized the distributions of correlations using the ForceAtlas2 algorithm in Gephi, and filtered the resulting graphs to exclude non-significant edges (i.e., correlations *p* > 0.05) from visualization (Bastian et al., 2009; Jacomy et al., 2014). There were visible differences between groups in functional network structure: ISO mice have fewer significant functional relationships than SOC mice, which is indicated by relatively few connections between nodes (Fig. 4*A–F*). For example, cFos-ir neurons in the PVN of ISO-FEN mice is significantly correlated with AH; thus, PVN shares an edge with only AH (Fig. 4*E*). Conversely, in SOC-FEN mice the number of cFos-ir neurons in the PVN is significantly correlated with LS, BNST, mPOA, AH, and PAG; thus, PVN shares edges with each of these regions (Fig. 4*B*). Further, the strength of functional relationships is indicated by the closeness of nodes in space. In SOC-pCPA mice, the significant correlation between cFos-ir in mPOA and BNST is indicated not only by a shared edge, but their relative adjacency (closeness) in space (Fig. 4*C*). In ISO-pCPA mice, there was a relatively weak, non-significant correlation between cFos-ir in mPOA and BNST which is reflected by a relatively large distance between these nodes in space (Fig. 4*F*).

We quantifiably described the network organization of the above graphs by performing three separate graph analyses. First, we quantified the number of significant functional relationships as a proportion of total possible functional relationships (i.e., functional density; Fornito et al., 2016). In each of the three SOC treatment groups, there was an over twofold increase in functional density compared with their ISO counterparts (Fig. 4*G*). While increased density indicates that there is more functional connectivity between regions in SOC mice, this metric tells us little about the nature of these correlations (Bullmore and Sporns, 2009). Importantly, do correlated nodes go on to form (1) additional functional relationships, and/or (2) larger functional modules?

Next, we calculated the clustering coefficient, the average number of connected pairs of a node’s connected neighbors of each treatment group. SOC mice had higher clustering coefficients than ISO mice in each treatment group (Fig. 4*H*). Indeed, regardless of drug treatment, ISO mice had a clustering coefficient of zero; thus, even when ISO mice have significant correlations between nodes, those regions do not in-turn form additional connections with each other. Our final graph analysis assessed modularity/community structure in SOC and ISO mice. In community structure analysis, the smallest number of communities that can be formed is one, indicating a completely connected group; the maximum number of communities is equal to the number of nodes contained in the analysis, and indicates complete functional segregation. We found that ISO mice formed more communities than their socially reared counterparts in each of the drug treatment groups (Fig. 4*I*). Together, our results support that functional networks are more disconnected in ISO mice.

## Discussion

Individual experience establishes the backdrop on which current events are interpreted. Early-life social isolation can profoundly affect an animal’s behavioral phenotype (Mumtaz et al., 2018), and likely affects how social signals (e.g., vocalizations) are represented in the brain; however, the effects of social isolation on the neural response to rodent vocalizations are relatively unexplored. We tested whether social experience interacts with serotonin signaling to affect IEG expression in the male SBN following playback of female BBVs. FEN robustly increased the number of cFos-ir neurons across all nodes of the SBN except PAG. Housing treatment also affected IEG induction: ISO mice had more cFos-ir neurons in several nodes of the SBN than SOC mice. Despite a generalized increase in cFos-ir, ISO mice had lower functional connectivity among regions than SOC mice. Indeed, functional density, clustering, and community structure remained relatively low in ISO mice despite pharmacological changes in available serotonin. Importantly, drug treatment had little effect on graph analyses in ISO mice and facilitated network measures in SOC mice.

### Social experience interacts with serotonin signaling to affect neural responses in the SBN

IEG mapping has established that early-life stressors alter neural activity markers at the level of individual SBN nodes. Chronic social subjugation decreased cFos expression in the LS, PVN, and PAG following open-arm exposure in male mice (Singewald et al., 2009). In rats, postweaning social isolation increased both aggression and cFos-ir neurons in the BNST and PVN (but not LS or PAG) following a resident-intruder paradigm (Toth et al., 2012). Despite structural interconnectedness, we found that changes in the cFos response in the SBN of ISO mice was not global: BNST, mPOA, PVN, VMH, and PAG had increased cFos-ir neurons, whereas LS and AH did not. Interestingly, the direction (i.e., an increase) of the cFos response was similar in each region affected in ISO mice.

The effects of housing on cFos-ir neurons were modulated by drug treatment. Similar to previous studies (Li and Rowland, 1993), we found that FEN increased cFos-ir neurons in all nodes of the SBN except for PAG. However, the effects of FEN were not homogenous across housing treatments: cFos-ir neurons were increased to a greater extent in the PVN and mPOA of ISO-FEN compared with SOC-FEN mice (Fig. 3*C*,*D*). Conversely, we found no difference in cFos-ir in the PVN of pCPA mice regardless of social experience, an observation consistent with previous studies in rats (Harbuz et al., 1993; Conde et al., 1998). Serotonin affects neural activity through a combination of excitatory and inhibitory serotonin receptor (5-HTR) subtypes (Barnes and Sharp, 1999), and 5-HTR expression patterns are sensitive to social isolation (Schiller et al., 2003; Bibancos et al., 2007); thus, the net effect of serotonergic manipulations is likely driven by complex excitatory and inhibitory interactions within and between each node of the SBN. Importantly, the increase in cFos-ir neurons was not observed exclusively in ISO-FEN mice: ISO-pCPA mice had more cFos-ir neurons in LS, BNST, mPOA, and PAG than SOC-pCPA mice.

Together, we found heterogenous effects of both pharmacology and housing conditions on the number of cFos-ir neurons within nodes of the SBN. An *a priori* assumption is that the SBN is a structurally interconnected network within which the patterns of functional connectivity are indicative of behavioral context (Newman, 1999; Goodson, 2005; Goodson and Kabelik, 2009). We therefore tested whether functional network measures differed within the SBN of SOC or ISO mice.

### Social experience affects functional connectivity in the SBN

The term functional connectivity, defined as “statistical dependencies among remote neurophysiological events” (Friston, 2011), has been used extensively in human fMRI studies to describe activity patterns in resting and pathologic states. This statistical phenomenon appears to be a crucial component to adaptive social processes across multiple species, including humans (Lynall et al., 2010). IEG mapping in non-traditional vertebrate model systems demonstrates functional connectivity between neuromodulatory systems/circuits (including the SBN) during vocal-acoustic processing in fishes (Petersen et al., 2013; Ghahramani et al., 2018) and frogs (Hoke et al., 2005), as well as prosocial and aggressive behavior in fishes (Weitekamp and Hofmann, 2017; Butler et al., 2018) and lizards (Kabelik et al., 2018). In male prairie voles (*Microtus ochrogaster*), oxytocin receptor antagonists reduce functional connectivity within the SBN and attenuate partner preference behavior (Johnson et al., 2016). In mice, Tanimizu et al. (2017) demonstrated that functional connectivity between memory-associated regions (including nodes of the SBN) was increased following a social learning task. Finally, different clusters of functional relationships in the SBN are observed in subordinate mice who maintain their beta status compared with those who ascend through the social hierarchy (Williamson et al., 2019).

We found that functional connectivity was decreased in ISO relative to SOC mice following playback of female BBVs. Importantly, these results were not because of a global increase in IEG induction as ISO mice tended to have more cFos-ir neurons than SOC mice. That functional connectivity is disrupted in the SBN of ISO mice represents an important foundation from which to develop models of how variation in functional network architecture relates to variation in aberrant behavioral (Keesom et al., 2017b; Manouze et al., 2019) and neural phenotypes (Keesom et al., 2017a, 2018) following early-life social stress.

We further analyzed patterns of functional connectivity by PCA and found that FEN mice had positive average PC1 scores, whereas SAL and pCPA mice had negative values (Fig. 4*I*). Thus, variation in the distribution of functional relationships differs following acutely increasing (i.e., with FEN) or systemically depleting (i.e., with pCPA) serotonin. Interestingly, the general direction of these relationships was consistent within drug treatment regardless of housing conditions. Therefore, while overall functional connectivity may be preferentially modulated by social experience, serotonin signaling drives variation in the nodes that are functionally coupled. However, the extent to which the effects of social experience and serotonin signaling are independent of each other remains unknown.

### Individual nodes disproportionately affect functional connectivity

PCA revealed that individual nodes disproportionately affect variation in functional connectivity. We found that eigenvectors within PC1 were most heavily loaded by mPOA and PVN. As mPOA and PVN underlie different functions, they may also drive variation in circuit-level metrics in different manners. mPOA has increased IEG induction following playback of social vocalizations in frogs and songbirds (Hoke et al., 2005; Maney et al., 2008). In mice, mPOA is a crucial site for affective-olfactory integration (Dhungel et al., 2011; McHenry et al., 2017), and activity in mPOA coincides with sociosexual investigation and facilitates mounting behavior (Wei et al., 2018). As the behavioral response to vocal signals (i.e., BBVs) is modulated by olfactory stimuli (Grimsley et al., 2013; Seagraves et al., 2016; Ronald et al., 2020), mPOA is in a functional anatomic position to integrate multisensory stimuli and effect circuit-level responses to vocal signals (Kohl et al., 2018). However, to our knowledge no studies have directly investigated mPOA involvement in rodent vocal processing.

We found that the number of cFos-ir neurons in the PVN is not only increased in ISO mice, but contributes a significant amount of variation to functional relationships within the SBN. Within the PVN, dysregulation of corticotropin-releasing factor (CRF) neurons which modulate the hypothalamic-pituitary-adrenal axis contributes to cardiovascular disease and impaired immune function in animal models of chronic social isolation as well as in persons with early-life social trauma (Heim and Nemeroff, 2001; McEwen, 2003; Cacioppo et al., 2015). Further, chemically heterogeneous PVN neurons underly different suites of behaviors: activating CRF neurons in PVN drives conditioned place aversion (Kim et al., 2019), whereas activating oxytocin neurons drives pup retrieval behavior in response to USVs (Marlin et al., 2015). Elucidating the chemical phenotypes and projection profiles of isolation-sensitive neurons in the PVN (and SBN in general) will be crucial to understanding the mechanisms through which different nodes affect functional relationships within the SBN.

### Network measures are lower in ISO relative to SOC mice

Variation in functional relationships includes reduced functional connectivity in ISO mice. Further, we found decreased network density and clustering in ISO mice, which is consistent with observations that similar reductions are observed in persons with schizophrenia (Lynall et al., 2010) and depression (Wise et al., 2017). Similarly, functional dysconnectivity within the SBN may provide a partial mechanism for decreased social competence observed in mice raised in social isolation (Keesom et al., 2017b). Investigating the mechanisms that drive variation in functional dysconnectivity will contribute to a better understanding of the diverse array of physiological and behavioral consequences observed in animals following social isolation and other early-life stressors (Heim and Nemeroff, 2001; Mumtaz et al., 2018). As neither FEN nor pCPA were sufficient to facilitate an increase in network measures in ISO mice, it is critically important to consider variation in an individual’s contextual state (i.e., social history, physiological condition, pathologic severity, etc.) when designing experiments and interpreting the results of psychoactive reagents on animal or human subjects (Bartz et al., 2011).

### Technical considerations

This study does not directly compare the cFos response in the SBN following playback of BBVs to a non-social acoustic stimulus (i.e., pure tones) or a silent condition. Playback of social vocalizations is sufficient to increase the cFos expression relative to tones in in the PAG of rats (Ouda et al., 2016), and relative to ambient environmental noise in multiple SBN nodes in the midshipman fish (*Porichthys notatus*; Petersen et al., 2013; Ghahramani et al., 2018). However, a lack of playback in the current study would not necessarily be a behaviorally neutral condition, given that silence could have a very different significance for socially housed versus isolated males. Likewise, mouse vocal signals encompass a wide range of frequencies and nonlinear structures (Holy and Guo, 2005; Grimsley et al., 2013; Lupanova and Egorova, 2015; Finton et al., 2017; Niemczura et al., 2020) and some commonly used ‘control’ sounds can evoke behavioral responses similar to call playback (K. Hood and L. Hurley, unpublished observations). In comparison to silence or other non-vocal sounds, playback of BBVs is a condition with a behavioral salience that is relatively well-understood. BBVs correspond to fewer male mounts of females in observational studies (Finton et al., 2017). Playback of BBVs alters the number of USVs as well as the numbers of males making USVs (Niemczura et al., 2020; Ronald et al., 2020). BBVs are therefore suppressive to some types of social behaviors, creating a defined behavioral context in the current study. However, we are unable to determine whether the observed cFos response in the SBN of SOC and ISO mice is selective for social vocalizations (Maney et al., 2006, 2008), whether functional connectivity is an emergent response to socially salient stimuli (Hoke et al., 2005; Ghahramani et al., 2018), or as a potential confound from a novel testing environment (VanElzakker et al., 2008) or as a direct result of social isolation in SOC animals (Matthews et al., 2016). Further studies are needed to directly test how the SBN responds to social vocalizations, and to probe the consequences of variation in functional connectivity in the production of murine social behaviors.

IEG mapping is limited in that there is no direct relationship with the production of action potentials and the presence of IEG products (including cFos; Clayton, 2000; Kovacs, 2008). Despite its wide use as a proxy for neural activation, the cFos protein is a transcription factor; one interpretation of our data is that instead of an increase in neural activity per se, we are observing an increased potential for neuroplasticity in ISO mice. Further, using cFos-ir as a putative marker for neural activity limits our temporal resolution to the entire 60-min trial. It is therefore impossible to make causal or directional statements pertaining to functional connectivity. We make no assumption that functional relationships imply structural connectivity, or vice versa. For example, a subset of mPOA neurons monosynaptically project to PVN (Kohl et al., 2018); however, the particular neurons that connect mPOA and PVN might not be similarly engaged in our paradigm.

Importantly, because the presence of cFos-ir is an indicator of past neural activity, it may be robust to action potentials that otherwise habituate over the course of a repeated stimulus i.e., auditory playback. For example, whereas neurons in the IC decrease spike rate following repeated presentation of auditory stimuli (Pérez‐González et al., 2005), cFos-ir neurons are detectable in the IC following 90 min playbacks of pure-tone stimuli (D’Alessandro and Harrison, 2014). Further, repeated presentation of ultrasonic distress calls increases cFos-ir within auditory and limbic structures in the rat (Ouda et al., 2016). We find it unlikely that the potential for SBN neurons to adapt to repeated playback of BBVs influenced our results. cFos takes ∼30 min to reach detectable changes in expression, and the half-life of the cFos protein is ∼45 min (Kovacs, 2008). The mice used in this study were at least 180 min removed from being transferred to the lab and 135 min removed from injections; we would expect cFos induction brought on by these aspects of our experimental design to be negligible.

Systemic pharmacological manipulations likely drive “off-target” effects such that activity markers are influenced not only by serotonin signaling within the SBN, but via direct or polysynaptic modulation of inputs into the SBN. Extrinsic to the SBN, depleting systemic serotonin reduces synaptic densities in the cortex of rats (Chen et al., 1994) and changes glutamate receptor distribution in the amygdala (Tran et al., 2013). Interestingly, functional connectivity/density, clustering, and community structure remain relatively high in SOC-FEN and SOC-pCPA mice despite potential off-target pharmacological and physiological effects (Otchy et al., 2015) resulting from chronically depleting or acutely increasing available serotonin. Manipulating site-specific serotonergic inputs into the SBN will be crucial to untangling the effects of individual nodes on network-level functionality (Ren et al., 2018). Importantly, network measures were also increased in SOC-SAL relative to ISO-SAL mice, suggesting the importance of social experience in the ability to form functional relationships in the SBN.

In conclusion, FEN and social isolation each broadly increased cFos-ir within individual nodes of the SBN following payback of social vocalizations. Importantly, by extending our analyses from individual nodes to the network level, we found that functional connectivity, clustering, and community structure within the SBN was highly dependent on social experience, whereas patterns of functional connectivity (i.e., which nodes formed functional relationships) were driven more by pharmacological manipulations. Our findings suggest the hypothesis that functional dysconnectivity may underlie psychopathological phenotypes that arise from social isolation and reinforces the importance to move beyond functional analyses limited to individual nodes. We highlight the importance of how laboratory housing conditions (SOC vs ISO) can affect functional neuroanatomical processes in rodents.

## Synthesis

Reviewing Editor: Elizabeth Cropper, Icahn School of Medicine at Mt. Sinai Decisions are customarily a result of the Reviewing Editor and the peer reviewers coming together and discussing their recommendations until a consensus is reached. When revisions are invited, a fact-based synthesis statement explaining their decision and outlining what is needed to prepare a revision will be listed below. The following reviewer(s) agreed to reveal their identity: NONE.

The authors have provided a thorough and thoughtful response and revision. They have addressed all of my concerns.
