# Supplementary material for: Social Experience Interacts with Serotonin to Affect Functional Connectivity in the Social Behavior Network following Playback of Social Vocalizations in Mice
Source: eNeuro. 2021 Mar 24;8(2):ENEURO.0247-20.2021. doi: 10.1523/ENEURO.0247-20.2021 (PMC8114900; doi:10.1523/ENEURO.0247-20.2021)
Supplement: Extended Data Figure 2-1 — Complete summary of post hoc analyses for linear mixed models performed within each SBN region. Download Figure 2-1, DOCX file. [file enu-eN-NWR-0247-20-s02.docx]

| Extended data table 2-1: Summary of post hoc analyses from linear mixed models | | | | | | |  |  |
| --- | --- | --- | --- | --- | --- | --- | --- | --- |
|  |  |  |  |  |  |  |  |  |
| **A: Main Effects of Housing** | | | | | | | | |
|  |  |  |  |  |  |  |  |  |
| Region | Housing | -Housing | Difference | Std Error | t | P | Lower 95% | Upper 95% |
| BNST | ISO | SOC | 3.87 | 1.44 | 2.69 | 0.02 | 0.79 | 6.94 |
| mPOA | ISO | SOC | 8.46 | 3.28 | 2.58 | 0.02 | 1.64 | 15.29 |
| PVN | ISO | SOC | 7.18 | 2.26 | 3.18 | 0.005 | 2.46 | 11.90 |
| PAG | ISO | SOC | 5.89 | 2.38 | 2.47 | 0.02 | 0.96 | 10.82 |

| **B: Main Effects of Drug Treatment** | | | | | | | | |
| --- | --- | --- | --- | --- | --- | --- | --- | --- |
|  |  |  |  |  |  |  |  |  |
| Region | Drug | -Drug | Difference | Std Error | t | P | Lower 95% | Upper 95% |
| Lateral Septum (LS) | FEN | pCPA | 8.30 | 1.68 | 4.96 | 0.0002 | 4.10 | 12.50 |
|  | FEN | SAL | 8.19 | 1.71 | 4.79 | 0.0002 | 3.91 | 12.47 |
|  | pCPA | SAL | -0.11 | 1.71 | -0.07 | 1.00 | -4.41 | 4.18 |
| Bed Nucleus of the Stria Terminalis (BNST) | FEN | pCPA | 15.44 | 1.74 | 8.85 | <.0001 | 10.89 | 19.99 |
|  | FEN | SAL | 15.68 | 1.77 | 8.87 | <.0001 | 11.07 | 20.29 |
|  | pCPA | SAL | 0.23 | 1.77 | 0.13 | 0.99 | -4.39 | 4.85 |
| Meidal Preoptic Area (mPOA) | FEN | pCPA | 24.47 | 4.01 | 6.10 | <.0001 | 14.34 | 34.59 |
|  | FEN | SAL | 25.06 | 4.02 | 6.23 | <.0001 | 14.90 | 35.21 |
|  | pCPA | SAL | 0.59 | 4.01 | 0.15 | 0.99 | -9.54 | 10.72 |
| Paraventricular Nucleus of the Hypothalamus (PVN) | FEN | pCPA | 21.71 | 2.75 | 7.89 | <.0001 | 14.72 | 28.69 |
|  | FEN | SAL | 21.49 | 2.77 | 7.77 | <.0001 | 14.46 | 28.51 |
|  | pCPA | SAL | -0.22 | 2.77 | -0.08 | 1.00 | -7.25 | 6.82 |
| Anterior Nucleus of the Hypothalamus (AH) | FEN | pCPA | 5.87 | 1.66 | 3.53 | 0.01 | 1.68 | 10.05 |
|  | FEN | SAL | 4.18 | 1.69 | 2.48 | 0.05 | -0.07 | 8.44 |
|  | pCPA | SAL | -1.68 | 1.69 | -0.99 | 0.59 | -5.94 | 2.58 |
| Ventromedial Nucleus of the Hypothalamus (VMH) | FEN | pCPA | 6.54 | 1.34 | 4.88 | 0.0002 | 3.17 | 9.92 |
|  | FEN | SAL | 6.03 | 1.37 | 4.40 | 0.001 | 2.58 | 9.48 |
|  | pCPA | SAL | -0.51 | 1.36 | -0.38 | 0.93 | -3.93 | 2.91 |

| **C: Housing*Drug Interaction** | | | | | | | |
| --- | --- | --- | --- | --- | --- | --- | --- |
|  |  |  |  |  |  |  |  |
| **Lateral Septum** |  |  |  |  |  |  |  |
| Treatment | -Treatment | Difference | Std Error | t Ratio | Prob>\|t\| | Lower 95% | Upper 95% |
| ISO-FEN | ISO-pCPA | 4.04 | 2.55 | 1.58 | 0.62 | -3.89 | 11.97 |
| ISO-FEN | ISO-SAL | 8.74 | 2.55 | 3.42 | 0.03 | 0.81 | 16.67 |
| ISO-FEN | SOC-FEN | -1.74 | 2.36 | -0.74 | 0.97 | -9.09 | 5.60 |
| ISO-FEN | SOC-pCPA | 10.82 | 2.37 | 4.56 | 0.002 | 3.45 | 18.19 |
| ISO-FEN | SOC-SAL | 5.89 | 2.47 | 2.39 | 0.20 | -1.77 | 13.56 |
| ISO-pCPA | ISO-SAL | 4.70 | 2.55 | 1.84 | 0.46 | -3.23 | 12.62 |
| ISO-pCPA | SOC-FEN | -5.79 | 2.36 | -2.45 | 0.18 | -13.13 | 1.56 |
| ISO-pCPA | SOC-pCPA | 6.78 | 2.37 | 2.86 | 0.08 | -0.59 | 14.15 |
| ISO-pCPA | SOC-SAL | 1.85 | 2.47 | 0.75 | 0.97 | -5.81 | 9.52 |
| ISO-SAL | SOC-FEN | -10.48 | 2.36 | -4.43 | 0.002 | -17.82 | -3.14 |
| ISO-SAL | SOC-pCPA | 2.08 | 2.37 | 0.88 | 0.95 | -5.29 | 9.45 |
| ISO-SAL | SOC-SAL | -2.84 | 2.47 | -1.15 | 0.85 | -10.51 | 4.82 |
| SOC-FEN | SOC-pCPA | 12.56 | 2.17 | 5.79 | <.0001 | 5.83 | 19.30 |
| SOC-FEN | SOC-SAL | 7.64 | 2.27 | 3.36 | 0.03 | 0.58 | 14.69 |
| SOC-pCPA | SOC-SAL | -4.93 | 2.28 | -2.16 | 0.30 | -12.01 | 2.16 |
|  |  |  |  |  |  |  |  |
| **Paraventricular Nucleus of the Hypothalamus** | | | |  |  |  |  |
| Housing | -Housing | Difference | Std Error | t Ratio | Prob>\|t\| | Lower 95% | Upper 95% |
| ISO-FEN | ISO-pCPA | 31.13 | 3.22 | 9.66 | <.0001 | 20.95 | 41.30 |
| ISO-FEN | ISO-SAL | 31.43 | 3.22 | 9.75 | <.0001 | 21.25 | 41.61 |
| ISO-FEN | SOC-FEN | 20.09 | 3.88 | 5.17 | 0.0007 | 7.82 | 32.35 |
| ISO-FEN | SOC-pCPA | 32.37 | 3.90 | 8.31 | <.0001 | 20.07 | 44.67 |
| ISO-FEN | SOC-SAL | 31.63 | 3.94 | 8.02 | <.0001 | 19.18 | 44.08 |
| ISO-pCPA | ISO-SAL | 0.30 | 3.22 | 0.09 | 1.00 | -9.87 | 10.48 |
| ISO-pCPA | SOC-FEN | -11.04 | 3.88 | -2.84 | 0.09 | -23.30 | 1.22 |
| ISO-pCPA | SOC-pCPA | 1.25 | 3.90 | 0.32 | 1.00 | -11.06 | 13.55 |
| ISO-pCPA | SOC-SAL | 0.51 | 3.94 | 0.13 | 1.00 | -11.94 | 12.95 |
| ISO-SAL | SOC-FEN | -11.34 | 3.88 | -2.92 | 0.08 | -23.61 | 0.92 |
| ISO-SAL | SOC-pCPA | 0.94 | 3.90 | 0.24 | 1.00 | -11.36 | 13.24 |
| ISO-SAL | SOC-SAL | 0.20 | 3.94 | 0.05 | 1.00 | -12.25 | 12.65 |
| SOC-FEN | SOC-pCPA | 12.29 | 4.46 | 2.76 | 0.11 | -1.79 | 26.36 |
| SOC-FEN | SOC-SAL | 11.55 | 4.50 | 2.57 | 0.15 | -2.66 | 25.75 |
| SOC-pCPA | SOC-SAL | -0.74 | 4.51 | -0.16 | 1.00 | -14.98 | 13.50 |
|  |  |  |  |  |  |  |  |
