# Supplementary material for: Social Experience Interacts with Serotonin to Affect Functional Connectivity in the Social Behavior Network following Playback of Social Vocalizations in Mice
Source: eNeuro. 2021 Mar 24;8(2):ENEURO.0247-20.2021. doi: 10.1523/ENEURO.0247-20.2021 (PMC8114900; doi:10.1523/ENEURO.0247-20.2021)
Supplement: Supplementary file 2 [file enu-eN-NWR-0247-20-s03.doc]

Social * Saline

	LS	BNST	PVN	AH	VMH	mPOA
**BNST**	r = 0.23<br>p = 0.58					
**PVN**	r = -0.05<br>p = 0.92	r = 0.25<br>p = 0.55				
**AH**	r = 0.06<br>p = 0.89	r = -0.28<br>p = 0.50	r = 0.39<br>p = 0.33			
**VMH**	r = 0.45<br>p = 0.31	r = 0.44<br>p = 0.32	r = 0.51<br>p = 0.24	**r = 0.77**<br>**p = 0.04**		
**mPOA**	r = 0.39<br>p = 0.33	r = 0.18<br>p = 0.68	r = 0.45<br>p = 0.26	*r = 0.67*<br>*p = 0.07*	**r = 0.89**<br>**p = 0.01**	
**PAG**	r = 0.33<br>p = 0.43	**r = 0.75**<br>**p = 0.03**	r = 0.35<br>p = 0.40	r = -0.12<br>p = 0.79	**r = 0.92**<br>**p = 0.003**	r = 0.53<br>p = 0.17

Isolated * Saline

	LS	BNST	PVN	AH	VMH	mPOA
**BNST**	*r = -0.59*<br>*p = 0.09*					
**PVN**	r = 0.06<br>p = 0.87	*r = 0.60*<br>*p = 0.09*				
**AH**	r = -0.13<br>p = 0.73	r = 0.51<br>p = 0.16	r = 0.54<br>p = 0.13			
**VMH**	r = 0.16<br>p = 0.74	r = -0.21<br>p = 0.66	r = 0.25<br>p = 0.58	r = 0.56<br>p = 0.19		
**mPOA**	r = -0.19<br>p = 0.62	*r = 0.63*<br>*p = 0.07*	r = 0.54<br>p = 0.13	**r = 0.84**<br>**p = 0.004**	r = 0.44<br>p = 0.32	
**PAG**	r = 0.44<br>p = 0.24	r = 0.03<br>p = 0.93	r = 0.45<br>p = 0.22	r = 0.36<br>p = 0.34	r = 0.28<br>p = 0.54	r = 0.48<br>p = 0.19

Social * Fenfluramine

	LS	BNST	PVN	AH	VMH	mPOA
**BNST**	**r = 0.90**<br>**p = 0.001**					
**PVN**	**r = 0.73**<br>**p = 0.02**	**r = 0.75**<br>**p = 0.02**				
**AH**	r = 0.46<br>p = 0.21	r = 0.35<br>p = 0.35	**r = 0.80**<br>**p = 0.01**			
**VMH**	r = 0.41<br>p = 0.31	r = 0.54<br>p = 0.16	r = 0.50<br>p = 0.21	r = 0.44<br>p = 0.28		
**mPOA**	r = 0.25<br>p = 0.54	r = 0.43<br>p = 0.29	**r = 0.75**<br>**p = 0.03**	r = 0.59<br>p = 0.13	**r = 0.75**<br>**p = 0.05**	
**PAG**	r = 0.40<br>p = 0.29	r = 0.57<br>p = 0.11	**r = 0.80**<br>**p = 0.01**	r = 0.54<br>p = 0.13	r = 0.32<br>p = 0.45	**r = 0.72**<br>**p = 0.05**

Isolated * Fenfluramine

	LS	BNST	PVN	AH	VMH	mPOA
**BNST**	r =0.48<br>p = 0.19					
**PVN**	r =-0.13<br>p = 0.73	r = 0.37<br>p = 0.32				
**AH**	r =0.05<br>p = 0.90	r = 0.31<br>p = 0.41	*r = 0.66*<br>*p = 0.06*			
**VMH**	r = -0.04<br>p = 0.93	r = 0.43<br>p = 0.29	r = 0.40<br>p = 0.33	r = 0.35<br>p = 0.40		
**mPOA**	r = 0.57<br>p = 0.11	r = 0.39<br>p = 0.29	r = 0.36<br>p = 0.34	r = 0.29<br>p = 0.45	r = 0.33<br>p = 0.42	
**PAG**	*r = 0.63*<br>*p = 0.07*	**r = 0.80**<br>**p = 0.01**	r = 0.21<br>p = 0.59	r = 0.23<br>p = 0.54	r = 0.41<br>p = 0.32	*r = 0.62*<br>*p = 0.07*

Social * pCPA

	LS	BNST	PVN	AH	VMH	mPOA
**BNST**	**r = 0.69**<br>**p = 0.04**					
**PVN**	**r = 0.83**<br>**p = 0.01**	r = 0.45<br>p = 0.23				
**AH**	r =0.30<br>p = 0.43	r = 0.20<br>p = 0.60	r = 0.43<br>p = 0.25			
**VMH**	*r = 0.64*<br>*p = 0.06*	r = 0.51<br>p = 0.16	**r =0.71**<br>**p = 0.03**	*r = 0.63*<br>*p = 0.07*		
**mPOA**	**r = 0.80**<br>**p = 0.01**	**r = 0.77**<br>**p = 0.02**	**r = 0.80**<br>**p = 0.01**	r = 0.21<br>p = 0.58	**r = 0.67**<br>**p = 0.05**	
**PAG**	r = 0.47<br>p = 0.20	**r = 0.70**<br>**p = 0.04**	r = 0.45<br>p = 0.23	r = -0.06<br>p = 0.88	r = 0.56<br>p = 0.12	**r = 0.81**<br>**p = 0.01**

Isolated * pCPA

	LS	BNST	PVN	AH	VMH	mPOA
**BNST**	r = 0.23<br>p = 0.55					
**PVN**	r = 0.30<br>p = 0.43	r = -0.01<br>p = 0.97				
**AH**	r = 0.60<br>p = 0.09	r = -0.12<br>p = 0.75	r = 0.55<br>p = 0.13			
**VMH**	r = 0.57<br>p = 0.11	r = 0.44<br>p = 0.23	**r = 0.80**<br>**p = 0.01**	r = 0.45<br>p = 0.23		
**mPOA**	r = 0.51<br>p = 0.16	r = -0.16<br>p = 0.67	r = -0.08<br>p = 0.83	**r = 0.71**<br>**p = 0.03**	r = -0.02<br>p = 0.97	
**PAG**	r = 0.58<br>p = 0.10	r = 0.35<br>p = 0.35	r = 0.60<br>p = 0.09	r = 0.57<br>p = 0.11	**r = 0.73**<br>**p = 0.02**	r = 0.40<br>p = 0.29
